# MicroRNA-19a-3p inhibits endothelial dysfunction in atherosclerosis by targeting JCAD

**DOI:** 10.1186/s12872-024-04063-y

**Published:** 2024-07-30

**Authors:** Jinque Luo, Ling Wang, Chaoyue Cui, Hongyu Chen, Wanli Zeng, Xin Li

**Affiliations:** 1 Hunan Provincial Key Laboratory of the Research and Development of Novel Pharmaceutical Preparations, “The 14th Five-Year Plan” Application Characteristic Discipline of Hunan Province (Pharmaceutical Science), College of Pharmacy, Changsha Medical University, 1501 Leifeng Avenue, Changsha, 410219 Hunan China; 2 College of Pharmacy, Hunan Provincial University Key Laboratory of the Fundamental and Clinical Research on Functional Nucleic Acid, Changsha Medical University, Changsha, 410219 Hunan China

**Keywords:** MicroRNA-19a-3p, Endothelial dysfunction, Inflammation, JCAD, Atherosclerosis

## Abstract

**Objective:**

To examine the influences and mechanisms of MicroRNA-19a-3p (miR-19a-3p) on endothelial dysfunction in atherosclerosis.

**Methods:**

An analysis of miR-19a expression was carried out using the Gene Expression Omnibus (GEO) database. The effect of miR-19a-3p on endothelial function in HUVECs was evaluated by miR-19a-3p overexpression under TNF-α treatment. Luciferase assays were performed to explore the potential target genes. Overexpression of junctional protein associated with coronary artery disease (JCAD) was used to examine the effects of miR-19a-3p on cell adhesion, and proliferation.

**Results:**

MiR-19a-3p expression in endothelial cells decreased after exposure to TNF-α and/or oscillatory flow, consistent with the expression change of miR-19a-3p found in atherosclerotic plaques. Additionally, endothelial cell dysfunction and inflammation were significantly diminished by miR-19a-3p overexpression but markedly exacerbated by miR-19a-3p inhibition. MiR-19a-3p transfection significantly decreased the expression of JCAD by binding to the 3’-UTR of JCAD mRNA. Furthermore, the protective effect of miR-19a-3p against endothelial cell dysfunction and inflammation was achieved by regulating JCAD and was closely linked to the Hippo/YAP signaling pathway.

**Conclusion:**

MiR-19a-3p expression is a crucial molecular switch in the onset of atherosclerosis and miR-19a-3p overexpression is a possible pharmacological therapeutic strategy for reversing the development of atherosclerosis.

**Supplementary Information:**

The online version contains supplementary material available at 10.1186/s12872-024-04063-y.

## Introduction

Atherosclerosis, a chronic inflammatory disease that underlies several important adverse vascular events, including myocardial infarction, coronary atherosclerosis, peripheral artery disease, and ischemic stroke, is responsible for most cardiovascular morbidity and mortality [1–4]. Fatty streaks in arterial walls are the early signs of atherosclerosis. Over time, these fatty streaks can develop into more advanced atheromatous plaques, the acute rupture of atheromatous plaques and subsequent thrombosis are major causes of cardiovascular events [5]. Each cell type can contribute to atherosclerosis at the tissue level, but the endothelium, a thin monolayer of cells covering the inner surface of blood channel walls, may be critical for the onset and progression of atherosclerosis [6–8]. Once endothelial cells become chronically activated by a combination of oscillatory flow, lipid accumulation, and proinflammatory cytokines (such as TNF-α and IL-1β) in the vessel wall, they promote the recruitment and maintenance of inflammatory cells into the subendothelial space, eventually leading to arterial wall thickening and atherosclerotic lesion formation [6, 9–11]. The functional integrity of the endothelium is the fundamental element for regulating vascular permeability and protecting vessels against atherosclerosis progression.

Endothelial dysfunction is the first step in the development of atherosclerosis and is caused by a variety of cardiovascular risk factors, including hypertension, hyperglycemia, and dyslipidemia [12–16]. When endothelial dysfunction occurs, there is a shift towards vasoconstriction, pro-inflammatory processes, and increased permeability to lipids and immune cells. These changes promote the initial stages of atherosclerosis, such as the accumulation of lipoproteins in the arterial wall and the migration of immune cells (like monocytes) into the subendothelial space [7, 13, 17]. Increasing data indicate that endothelial cell dysfunction is distinguished by elevated inflammatory responses within the blood vessel wall and perturbed vascular tone and redox balance [18–21]. Inflammation caused by risk factors such as oscillatory flow and oxidized LDL leads to increased production of IL-1, IL-6, TNF-α, and CRP, resulting in endothelial cell activation and dysfunction with increased expression of adhesion molecules that trigger leukocyte homing, adhesion, and migration into the subendothelial space [12, 22]. Endothelial function regulation involves the precise coordination of molecular and cellular activities influenced by stimulating and inhibitory signals that lead to a controlled physiological response. As such, highly dynamic and dose-sensitive signaling complexes are prime candidates for microRNA (miR) posttranscriptional-mediated regulation of gene expression programs in endothelial cells [23]. Understanding the role of miR in endothelial function regulation is pivotal for unraveling their profound impact on vascular health and disease.

MiRs are endogenously expressed small noncoding RNAs that are specifically expressed by certain organs or cell types; they act as transcriptional and posttranscriptional regulators of gene expression and play essential roles in the regulation of cell biology processes [24, 25]. To date, approximately 2600 miRs have been identified in humans, but for many, there is still a lack of sufficient research on their biological activities [26]. In recent years, multiple reports have suggested that miRs could be regarded as novel biomarkers for cardiovascular diseases, including atherosclerosis, diabetes mellitus, acute myocardial infarction, and stroke [27–29]. For instance, miR-126 and miR-143/145 play crucial roles in the regulation of cardiovascular development and differentiation [30, 31], and miR-21 potentially contributes to the enlargement of the necrotic core in atherosclerosis [32]. Furthermore, due to their key role in regulating the gene expression of endothelial cells, miRs have been shown to be important modulators of endothelium homeostasis in cardiovascular disease. An increasing number of studies point to the involvement of miRs, such as miR-1 and miR-210, in the control of endothelial cell function, cytokine response, and vascular inflammation in atherosclerosis [29, 33–35]. Understanding the molecular mechanisms underlying endothelial dysfunction, including the role of miRs, is essential for identifying new therapeutic targets and developing strategies to prevent or treat atherosclerosis.

MiRs, as important members of the single-stranded, small noncoding RNA family, can bind to the 3’-untranslated region (3’-UTR) of target messenger RNA (mRNA) sequences, reducing gene expression by inducing mRNA degradation or blocking translation [36, 37]. MiRs act either by themselves or in clusters; when miRs in a cluster are coexpressed to control numerous target genes, they generally work together, but they typically do not have similar sequences or target the same genes [38]. One of the most well-known miR clusters, miR-17-92, has been identified as an oncogene and consists of miR-17, miR-18a, miR-19a/b, miR-20a, and miR-92a [39]. MiR-17 inhibition has been shown to mitigate atherosclerosis by decreasing inflammation and lipid accumulation in atherosclerotic lesions [40]. MiR-20a/b and miR-92a act as regulators of lipid metabolism [41]. However, the changes in the expression and regulation of miR-19a-3p in cardiovascular disease are still controversial, with some researchers observing an increase and others finding no change or even decreased expression [42–44]. Similarly, there is controversy about the alterations in the level of miR-19a-3p in endothelial blood vessel cells; both atherogenic hypoxia-inducible factors and atheroprotective unidirectional laminar flow have the same impact on miR-19a-3p levels [45, 46]. Thus, the role of miR-19a-3p in cardiovascular disease still needs to be further investigated.

In this study, we found that miR-19a-3p levels were lower in the tissues of mice with atherosclerotic coronary artery disease than in the tissues of control mice and identified junctional proteins associated with coronary artery disease (JCAD) as a new target for miR-19a-3p. As a gene associated with coronary artery disease, JCAD may promote endothelial dysfunction and atherosclerosis [47]. MiR-19a-3p suppressed JCAD expression and regulated the Hippo/YAP signaling pathway in endothelial cells. In addition, the protective effect of miR-19a-3p against endothelial cell dysfunction was achieved by regulating JCAD. According to these findings, miR-19a-3p functions as an atheroprotective miR during the progression of atherosclerosis.

## Materials and methods

### Bioinformatics analysis

The GSE34645, GSE34646, and GSE26953 miR datasets were used in this study. The Limma package (R language) was used for intergroup difference analysis with |logFC|> log_2_1.5 and *P* < 0.05 as the cutoffs for GSE34645. The Limma package (R language) was applied for intergroup difference analysis with |logFC|> log_2_2 and *P* < 0.05 as the cutoffs for GSE34646 and GSE26953. A heatmap was generated to visualize the various miR analysis results.

### Cell culture

Primary human umbilical vein endothelial cells (HUVECs) were isolated from fresh human umbilical cords following bioethical guidelines. The cells were then cultured in Medium 200 containing 5% FBS and 1× low serum growth supplement (LSGS) (Invitrogen, CA, USA) following a previously described protocol [47]. THP-1 cells (ATCC, VA, USA) were cultured in RPMI 1640 medium supplemented with 10% FBS. HEK-293T (ATCC, VA, USA) cells were grown in high-glucose DMEM supplemented with 10% FBS. All cells were cultured at 37 °C in a humidified atmosphere of 5% CO_2_.

### Treatment of HUVECs

HUVECs at 80% confluence were treated with 10 ng/mL TNF-α (MCE, Shanghai, China) for 24 h. For steady laminar flow experiments, HUVECs cultured in fresh complete media were exposed to a laminar flow (12 dyn/cm^2^) in a 35 mm cone and plate viscometer [47] for various durations.

### Transfection with miRNAs

Human miR-19a-3p mimics (UGUGCAAAUCUAUGCAAAACUGA), miR-19a-3p antisense oligonucleotide (anti-miR-19a-3p, UCAGUUUUGCAUAGAUUUGCACA), and control oligonucleotides (miR-c or negative anti-miR) were obtained from GenePharma Co., Ltd. (Shanghai, China) and transfected into HUVECs at a final concentration of 50 pmol/mL using Lipofectamine 3000 (Invitrogen, CA, USA) according to the manufacturer’s protocol.

### Adenovirus-mediated overexpression of JCAD

In the overexpression experiment, at 80% confluence, HUVECs were infected with control adenovirus (Control) or JCAD adenovirus (Ad-JCAD, M.O.I.=2, #EX-H2031-M08, GeneCopoeia; virus custom made by Abm Good Inc., Canada) for 24 h.

### Real-time quantitative PCR (qRT‒PCR)

Total RNA, including miRs, was isolated using TRIzol reagent (Invitrogen; Thermo Fisher Scientific, USA) following the manufacturer’s instructions [48]. RT‒qPCR was used to measure relative miR-19a-3p expression using the miDETECT A Track miRNA qRT‒PCR Starter Kit (RiboBio, Guangzhou, China) following the manufacturer’s instructions. Primers and other reagents for miR assays were purchased from RiboBio, and the data were normalized to the expression level of U6 [49]. The relative mRNA expression of other genes was measured by qRT‒PCR using a standard SYBR-Green RT‒PCR kit (Takara, Dalian, China). GAPDH was used as a control for the genes, and the 2^−ΔΔCT^ method was used to obtain the relative expression levels. The primer sequences are detailed in Table S1.

### Dual-luciferase reporter gene assay

The wild-type (WT) fragment of the JCAD 3’-UTR containing the miR-19a-3p binding sequence was cloned and inserted into the pmirGLO vector (Promega, Madison, WI, USA) to construct JCAD-WT. Then, the target-binding sequence between JCAD and miR-19a-3p was mutated, and the mutant (MUT) fragment was cloned and inserted into the pmirGLO vector to construct JCAD-MUT. JCAD-WT or JCAD-MUT and miR-19a-3p mimics or miR-c were cotransfected into HUVECs. 48 h later, luciferase activity was assessed with a dual-luciferase reporter gene detection system (Promega, Madison, WI, USA) according to the manufacturer’s protocol.

### In vitro monocyte adhesion assay

Monocyte adhesion to endothelial cells was determined using THP-1 monocytes as previously described [47]. In brief, HUVECs were pretreated with either miR-19a-3p (50 pmol/mL) or anti-miR-19a-3p (50 pmol/mL) for 24 h before they were stimulated with TNF-α or laminar flow. Then, THP-1 monocytes were added to the HUVEC monolayers and incubated for an additional 30 min. Nonadherent THP-1 cells were removed by washing three times with prewarmed serum-free high glucose DMEM. The attached cells were then observed under an inverted Zeiss Axiovert 40 C microscope (magnification: ×10; numeric aperture: 0.25; Carl Zeiss) and photographed using a Canon A640 digital camera. The number of monocytes attached to endothelial cells was manually calculated.

### Immunofluorescence staining and confocal microscopy

HUVECs were plated in 35 mm glass-bottom dishes at 90% confluence. After being treated with miR-19a-3p for 24 h, the cells were fixed with 4% paraformaldehyde (PFA) in phosphate-buffered saline (PBS) for 15 min and then washed three times with PBS. The cells were treated with 0.1% Triton X-100 in PBS for an additional 15 min at room temperature to permeabilize the cell membrane. Afterward, the cells were blocked with 10% goat-blocking serum from Thermo Fisher Scientific before incubation with primary antibodies against YAP/TAZ (CST, MA, USA) at 4 °C overnight. Then, the cells were washed and incubated with the appropriate secondary antibodies in a blocking reagent for 1 h at room temperature. After rinsing with PBS, the nuclei were stained with DAPI in PBS for 15 min. The stained cells were mounted with ProLong Gold-anti-fade mounting media (Thermo Fisher Scientific) and covered with glass coverslips. All images were taken with a Zeiss (Jena, Germany) LSM800 confocal microscope.

### Western blotting assay

Protein lysates from treated HUVECs were prepared following standard protocols, and the protein concentration was measured using a BCA protein assay kit (Thermo Fisher Scientific) [50, 51]. All protein samples were separated by SDS‒PAGE, and proteins were transferred onto PVDF membranes (Millipore, Burlington, MA, USA). The membranes were blocked in 5% bovine serum albumin (BSA, Sigma‒Aldrich) in TBST (Tris-buffered saline, 0.1% Tween 20) for 1 h at room temperature and incubated with different primary antibodies overnight at 4 °C [52]. The membranes were washed three times and incubated with secondary antibodies for 1 h at room temperature. Protein bands were detected with an enhanced chemiluminescence reaction kit (Millipore, Burlington, MA, USA) on a chemiluminescence imaging system (Tanon 5200, Yuanpinghao Biotechnology, Beijing, China) and analyzed using ImageJ software. The details of the sources of the primary antibodies are provided in Table S2.

### Cell counting Kit-8 (CCK8)

Cells were digested, counted, and seeded into a 96-well plate at a density of 5 × 10^4^ cells/well (100 µL/well). Three replicate wells were used for each group. After culture and adhesion, the CCK8 solution (10:1, NU679, DOJINDO) was prepared with a complete medium and then added to each well as reported [53]. A Bio-Tek microplate (MB-530, Heals) was used to analyze the absorbance at 450 nm.

### Statistical analysis

The data are presented as the means ± SD unless otherwise indicated. Statistical analysis was performed with Student’s t test for two groups and one-way or two-way ANOVA for multiple groups with GraphPad Prism. P values less than 0.05 (*P* < 0.05) were considered to indicate statistical significance.

## Results

### MiR-19a expression is downregulated in atherosclerosis

**Fig. 1:**
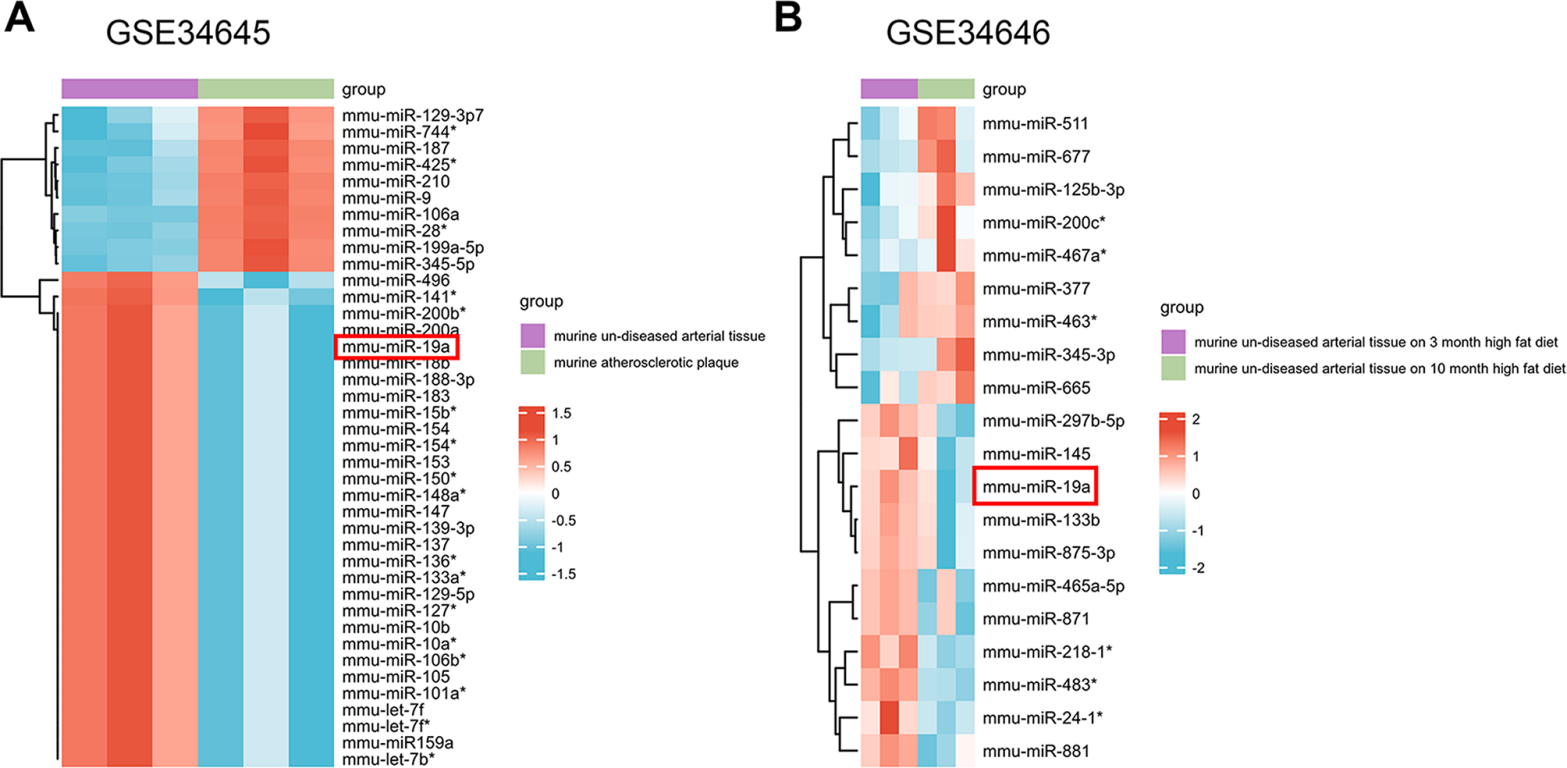
The miR-19a level decreases as atherosclerosis progresses. (**A**) A heatmap was generated to show the top 40 differentially expressed miRs between atherosclerotic plaques and nondiseased arterial tissue. The differentially expressed miRs were identified using the GEO chip GSE34645 with |logFC| > 1.5 and adj *P* < 0.05 as the screening criteria. (**B**) A heatmap was generated to show the top 20 differentially expressed miRs between arterial tissue from mice fed a high-fat diet for 3 months and mice fed a high-fat diet for 10 months. The differentially expressed miRs were identified using the GEO chip GSE34646 with a |logFC| > 2 and adj *P* < 0.05 as the screening criteria

To investigate the role of miR signatures in the progression of atherosclerosis, we downloaded and analyzed the GSE34645 and GSE34646 datasets from the GEO database. Differentially expressed miRs were selected from the GEO chip GSE34645 under the screening criteria |log_2_(FoldChange)| > 1.5) and adj *P* < 0.05. A heatmap for the top 40 miRs is presented in Fig. 1A, which suggested that after 3 months on a high-fat diet, miR-19a expression was lower in atherosclerotic plaques than in healthy artery tissue in mice. Additionally, the top 20 differentially expressed miRs were identified according to multiple differences (|log_2_(FoldChange)| > 2) and significance of expression differences (*P*-adj < 0.05) in the GEO chip GSE34646; their expression values are shown in a heatmap (Fig. 1B). MiR-19a expression was lower in arterial tissue from mice fed a high-fat diet for 10 months than in arterial tissue from mice fed a high-fat diet for 3 months. Based on these findings, we speculated that miR-19a might be a new potential therapeutic target for the prevention of atherosclerosis progression.

### Upregulation of miR-19a-3p expression in endothelial cells is atheroprotective

**Fig. 2:**
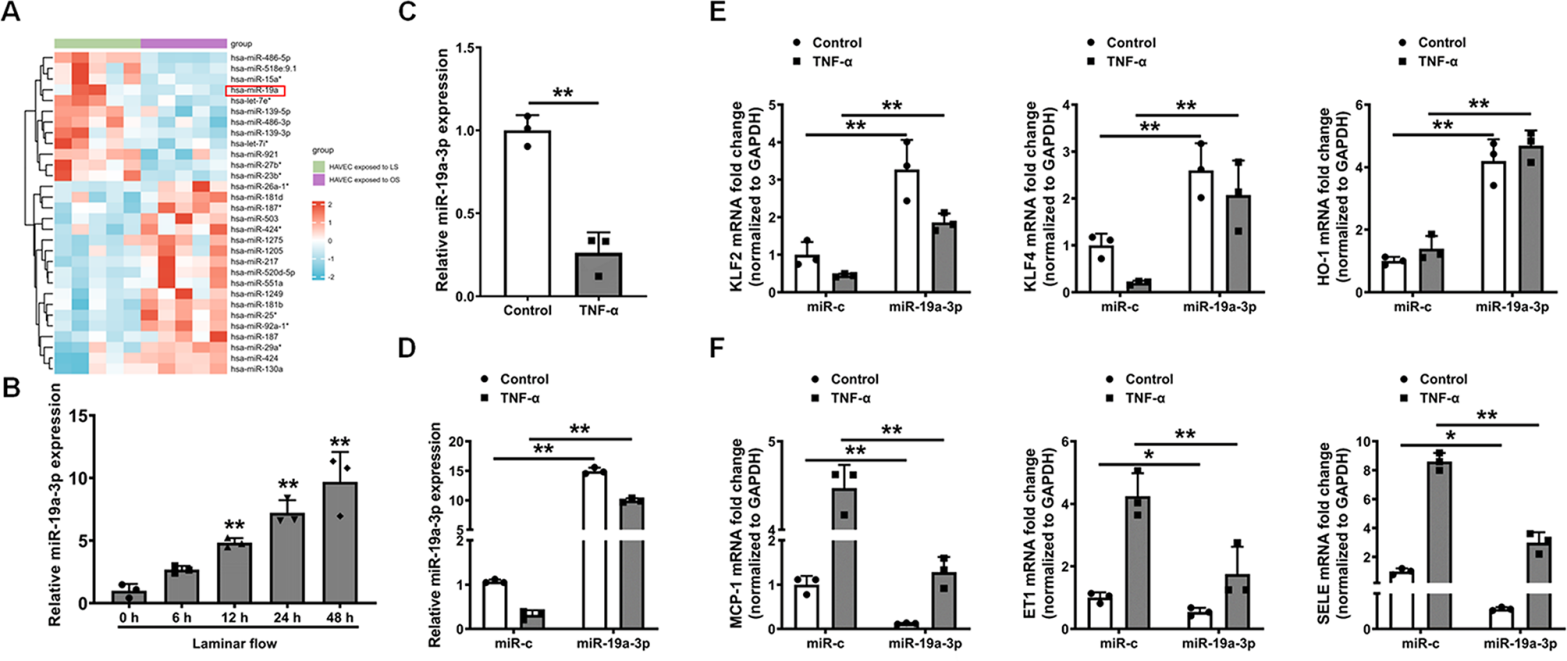
The overexpression of miR-19a-3p in endothelial cells is atheroprotective. (**A**) A heatmap was generated to show the differentially expressed miRs in human aortic valve endothelial cells (HAVECs) after 24 h of oscillatory flow or laminar flow exposure. (**B**) Expression level of miR-19a-3p in HUVECs exposed to 12 dyn/cm^2^ laminar shear stress for various durations. The U6 snRNA level was used for normalization. The data were measured by qRT‒PCR and presented as means ± SD (*n* = 3). **P < 0.01 compared with the static control (ANOVA). (**C**) Changes in the expression of miR-19a-3p in HUVECs after treatment with or without TNF-α (10 ng/mL) for 24 h. The data are presented as means ± SD (*n* = 3). **P < 0.01 (Student’s t test). (**D**) Changes in miR-19a-3p levels in HUVECs at 24 h after transfection with miR-c and the miR-19a-3p mimic in the presence or absence of 10 ng/mL TNF-α. (**E**,** F**) Treatment of HUVECs with miR-19a-3p (50 pmol/mL) significantly upregulated the expression of atheroprotective genes (KLF2, KLF4, and HO-1) but downregulated the expression of atheropromoting genes (MCP-1, ET1, and SELE) in HUVECs under different conditions (in the presence or absence of 10 ng/mL TNF-α). The data were obtained by qRT‒PCR and presented as means ± SD (*n* = 3). *P < 0.05, **P < 0.01 (ANOVA)

To assess how athero-relevant treatments regulate the expression of miR-19a-3p, we first evaluated the miR expression profiles of human aortic valve endothelial cells (HAVECs) after 24 h of oscillatory flow or laminar flow exposure by mining the GSE26953 dataset from the GEO database. Oscillatory flow is a nonunidirectional flow that mimics conditions in atherosclerosis-prone regions of arterial circulation [48]. Laminar flow is a recognized representation of “atheroprotective” flow conditions. Among the miRs represented on the microarrays, the expression of 12 miRs was considerably lower and that of 18 was significantly greater in the laminar flow-treated HAVECs than in the oscillatory flow-treated HAVECs. Compared to atheroprotective laminar flow, miR-19a was expressed at a significantly lower level in atheropromoting oscillatory circumstances (Fig. 2A). This result indicates that low expression of miR-19a in endothelial cells may increase the risk of atherosclerosis.

Next, HUVECs were subjected to laminar shear stress for various periods to analyze the temporal dynamics of miR-19a-3p expression. We found that miR-19a-3p was a shear stress early responsive gene in endothelial cells, as shown by qRT‒PCR analysis, the results of which indicated a large increase in miR-19a-3p expression as early as 12 h after shear treatment (Fig. 2B), which is consistent with the findings of other studies [36]. TNF-α contributes to endothelial cell dysfunction in several key ways, which are pivotal in the early stages of atherosclerosis. When HUVECs were treated with TNF-α, miR-19a-3p expression was significantly downregulated (Fig. 2C). These findings suggest that pro-atherogenic treatments have the potential to drastically reduce miR-19a-3p expression, making miR-19a-3p a new potential therapeutic target for the treatment of atherosclerosis. Further research into the precise role of miR-19a-3p in atherosclerosis and endothelial cell function is essential.

To determine the influence of miR-19a-3p on endothelial cell function, we performed a gene expression assay in TNF-α-treated HUVECs. HUVECs were transfected with a miR-19a-3p mimic, which increased the expression of miR-19a-3p both with and without TNF-α (Fig. 2D). The overexpression of miR-19a-3p had no effect on the expression of other miRs (Fig. S1), the expression of which was downregulated by TNF-α treatment in HUVECs, as reported by Tang and colleagues [54]. Our data indicate that the overexpression of miR-19a-3p increased the expression of the atheroprotective genes Kruppel-like factor 2 (KLF2), Kruppel-like factor 4 (KLF4), and heme oxygenase 1 (HO-1), decreased the expression of the proatherogenic genes monocyte chemoattractant protein-1 (MCP-1), endothelin-1 (ET1), and SELE in the presence or absence of TNF-α (Fig. 2E and F). These data suggest that the overexpression of miR-19a-3p may render endothelial cells homeostatic (anti-inflammatory, antioxidative, and antithrombotic) and thus may confer protection against endothelial dysfunction.

### MiR-19a-3p overexpression inhibits endothelial dysfunction

**Fig. 3:**
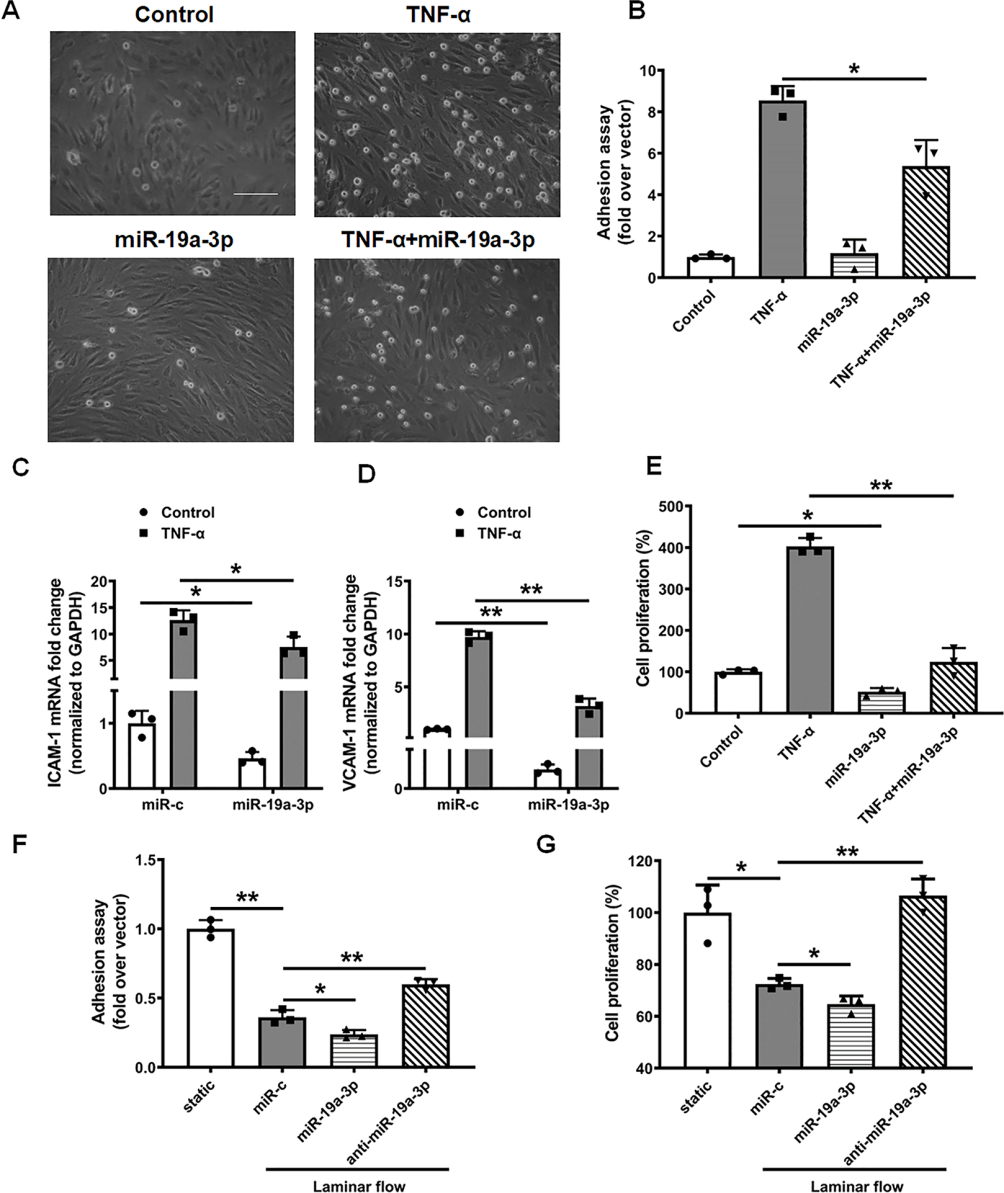
The overexpression of miR-19a-3p inhibits endothelial dysfunction. (**A, B**) MiR-19a-3p reduces TNF-α-induced cell adhesion in HUVECs. (**A**) Effect of miR-19a-3p on monocyte adhesion to endothelial cells. HUVECs were treated with miR-19a-3p (50 pmol/mL) in the presence or absence of TNF-α (10 ng/mL) for 24 h before THP-1 monocytes were added. Scale bar = 200 μm. (**B**) Bar diagram showing the number of adherent THP-1 monocytes in (A). The data are presented as means ± SD (*n* = 3). (**C**, **D**) The relative mRNA levels of ICAM-1 and VCAM-1 in HUVECs treated with miR-19a-3p (50 pmol/mL) for 24 h were measured by qRT‒PCR. The data are presented as means ± SD (*n* = 3). (**E**) Treatment with miR-19a-3p (50 pmol/mL) for 24 h inhibited TNF-α-stimulated HUVEC proliferation, as determined by a CCK-8 assay. The data are presented as means ± SD (*n* = 3). (**F**) THP-1 monocyte adhesion assay was conducted with HUVECs. HUVECs were pretreated with either miR-19a-3p (50 pmol/mL) or anti-miR-19a-3p (50 pmol/mL) for 24 h before they were treated with or without laminar flow for an additional 24 h. Then, THP-1 monocytes were added to monolayers of HUVECs and incubated for an additional 30 min. The number of monocytes attached to endothelial cells was manually calculated. The data are presented as means ± SD (*n* = 3). (**G**) CCK-8 assay was conducted with HUVECs subjected to 24 h of laminar shear (12 dynes/cm^2^) after treatment with either miR-19a-3p (50 pmol/mL) or anti-miR-19a-3p (50 pmol/mL). The data are presented as means ± SD (*n* = 3). *P < 0.05, **P < 0.01 (ANOVA)

Endothelial dysfunction is characterized by vasoconstriction, cell proliferation, and a shift toward a proinflammatory and prothrombic state, which is a major contributor to the development of cardiovascular diseases, including atherosclerosis [55]. To investigate the function of miR-19a-3p in endothelial function and atherosclerosis, we assessed whether miR-19a-3p regulates monocyte adhesion to HUVECs, a key step in the initiation and progression of atherosclerosis. Treatment with the miR-19a-3p mimic reduced THP-1 monocyte adhesion to HUVECs in response to TNF-α (Fig. 3A and B), which was associated with decreased expression of the proinflammatory adhesion molecules vascular cell adhesion molecule 1 (VCAM-1) and intracellular adhesion molecule 1 (ICAM-1) (Fig. 3C and D). In addition, the miR-19a-3p mimic significantly decreased the proliferation of HUVECs treated with or without TNF-α (Fig. 3E). Endothelial proliferation in blood arteries can lead to sprouting angiogenesis, which may exacerbate plaque instability in atherosclerosis [56]. Together, these results demonstrate the protective role of miR-19a-3p in endothelial cell activation and inflammation in atherosclerosis.

Additionally, miR-19a-3p mimic treatment inhibited THP-1 monocyte adhesion and cellular proliferation in HUVECs under laminar shear stress conditions (Fig. 3F and G), whereas anti-miR-19a-3p-mediated knockdown of miR-19a-3p (Fig. S2) significantly increased THP-1 monocyte adhesion and cellular proliferation in HUVECs under laminar shear stress conditions (Fig. 3F and G). These results further demonstrate the protective role of miR-19a-3p in endothelial dysfunction.

### MiR-19a-3p regulates JCAD expression by targeting its 3’-UTR

**Fig. 4:**
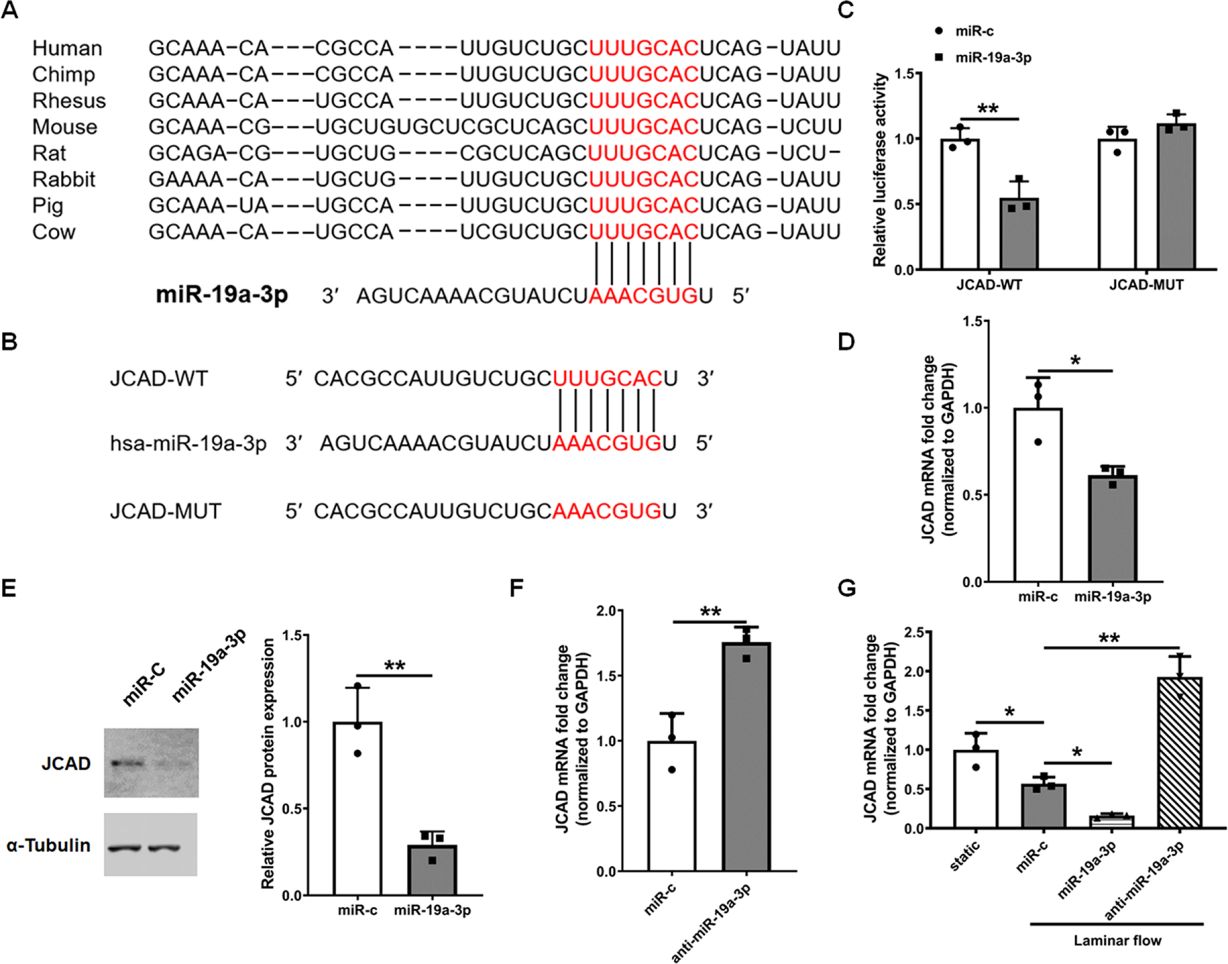
MiR-19a-3p can regulate JCAD by targeting its 3’-UTR. (**A**) “Seed” sequences at miR-19a-3p binding sites and complementary bases in the JCAD 3’-UTR are shown for humans and other species. (**B**) Wild-type (WT) and mutated (MUT) binding sites for miR-19a-3p and the JCAD 3’-UTR in humans is shown. (**C**) The activity of firefly luciferase was analyzed after cotransfecting the JCAD-WT or JCAD-MUT reporter plasmids into HEK293 cells infected with the miR-19a-3p mimic. Luciferase activity was normalized to Renilla’s activity. The data are presented as means ± SD (*n* = 3), **P < 0.01 (ANOVA). (**D**) MiR-19a-3p can inhibit the mRNA expression of JCAD in HUVECs. HUVECs were treated with or without miR-19a-3p (50 pmol/mL) for 24 h, and the mRNA expression of JCAD was obtained by qRT‒PCR and presented as means ± SD (*n* = 3), *P < 0.05 (Student’s t test). (**E**) MiR-19a-3p can inhibit the protein expression of JCAD in HUVECs. HUVECs were treated with or without miR-19a-3p (50 pmol/mL) for 24 h, the protein expression of JCAD was obtained by western blotting and normalized to GAPDH, and the results are presented as means ± SD (*n* = 3). *P < 0.05 (Student’s t test). (**F**) Anti-miR-19a-3p can increase the mRNA expression of JCAD in HUVECs. HUVECs were treated with or without anti-miR-19a-3p (50 pmol/mL) for 24 h, the mRNA expression of JCAD was obtained by qRT‒PCR and presented as means ± SD (*n* = 3), **P < 0.01 (Student’s t test). (**G**) Anti-miR-19a-3p prevented the inhibitory effect of laminar flow on JCAD expression. HUVECs were pretreated with anti-miR-19a-3p (50 pmol/mL) or control oligonucleotides (negative anti-miR) for 24 h followed by laminar flow (12 dynes/cm^2^) for 24 h, and JCAD expression was examined via qRT‒PCR. The results are presented as the mean ± SD (*n* = 3). *P < 0.05, **P < 0.01 (ANOVA)

To elucidate the mechanism of action of miR-19a-3p in endothelial dysfunction, we used TargetScan (Release8.0, http://www.targetscan.org/) to identify the downstream target of miR-19a-3p and found a putative binding site in the 3’-UTR of JCAD mRNA to the seed sequence of miR-19a-3p, which is highly conserved across numerous species (Fig. 4A). We generated mutants for this site and performed a luciferase reporter assay in HEK-293T cells by transfecting a miR-19a-3p mimic and a luciferase reporter downstream with either the wild-type JCAD 3’-UTR or a mutated JCAD 3’-UTR (Fig. 4B). With the miR-19a-3p mimic, we found that miR-19a-3p suppressed reporter activity and that the site mutation reversed this suppression (Fig. 4C). These results suggest that JCAD is a direct target of miR-19a-3p and that miR-19a-3p regulates JCAD expression by directly targeting specific binding sites in the 3’-UTR of JCAD.

The qRT‒PCR and western blotting results suggested that miR-19a-3p overexpression significantly reduced JCAD expression in HUVECs (Fig. 4D, E). As a novel coronary artery disease gene, JCAD could promote atherosclerotic plaque formation via a role in the endothelial cell shear stress mechanotransduction pathway. By influencing endothelial responses to mechanical forces, JCAD contributes to endothelial dysfunction, inflammation, and plaque instability. Targeting JCAD or its downstream pathways holds promise for developing novel treatments for coronary artery disease and atherosclerosis [47]. To study the effect of miR-19a-3p on laminar shear-treated endothelial cells, anti-miR-19a-3p was introduced into HUVECs under laminar shear stress. The inhibition of endogenous miR-19a-3p with anti-miR-19a-3p (Fig. 4F) attenuated the suppressive effects of 24 h of laminar flow on the JCAD level, thus confirming that the shear inhibition of JCAD protein expression is under the control of miR-19a-3p (Fig. 4G).

### MiR-19a-3p alters the Hippo/YAP signaling pathway

**Fig. 5:**
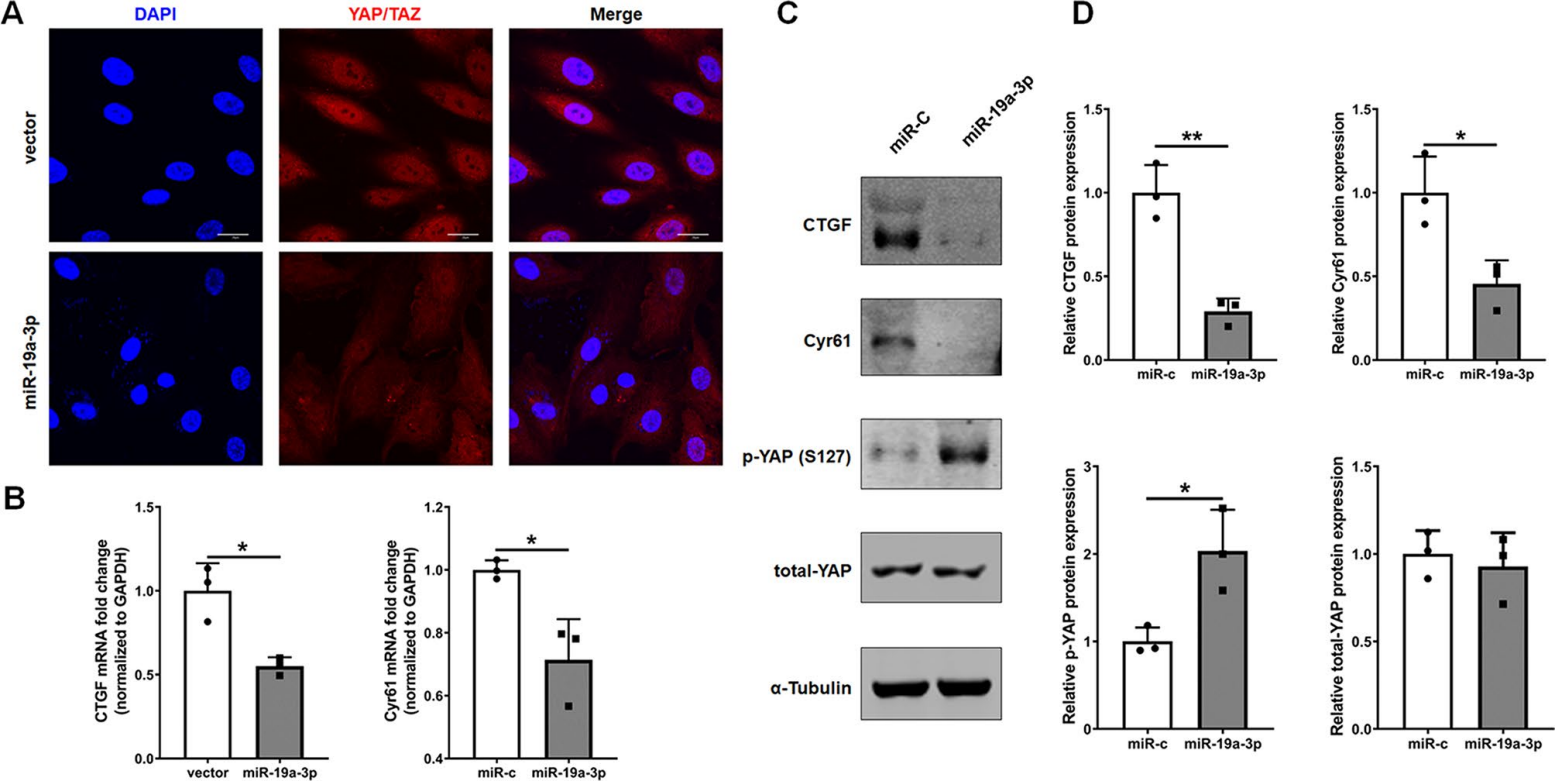
Overexpression of miR-19a-3p inhibits YAP/TAZ activity. (**A**) MiR-19a-3p (50 pmol/mL) treatment decreased YAP/TAZ nuclear translocation (red), as shown by immunofluorescence staining. DAPI was used to counterstain nuclei, and the scale bar corresponds to 50 μm, *n* = 3. (**B**) MiR-19a-3p (50 pmol/mL) treatment decreased the mRNA expression of the YAP/TAZ downstream genes CTGF and Cyr61. The data are presented as means ± SD (*n* = 3). (**C**, **D**) MiR-19a-3p (50 pmol/mL) treatment increased YAP phosphorylation and decreased the expression of the YAP/TAZ downstream proteins CTGF and Cyr61. The data are presented as means ± SD (*n* = 3). *P < 0.05, **P < 0.01 (Student’s t test)

Previous studies have shown that the Hippo/YAP pathway regulates endothelial cell cycle progression as well as vascular inflammation, by modulating these processes, this pathway significantly influences endothelial function and the development of atherosclerosis [57, 58]. The YAP downstream genes CTGF and Cyr61, whose expression is downregulated by JCAD depletion, were also involved in endothelial function, mechanotransduction pathways, and vascular health [47, 59]. We believe that miR-19a-3p may inhibit YAP activation in endothelial cells. To this end, we first investigated how miR-19a-3p affects the nuclear staining of YAP/TAZ in endothelial cells. We found that overexpressing miR-19a-3p dramatically reduced YAP/TAZ nuclear staining (Fig. 5A). Consistent with these results, we observed that YAP phosphorylation is increased, resulting in its downstream targets, including CTGF and Cyr61, was significantly decreased in miR-19a-3p-overexpressing HUVECs (Fig. 5B-D). These findings suggest that miR-19a-3p could inhibit endothelial cell activation by inhibiting YAP activation, which underscores its potential as a therapeutic target for atherosclerosis.

### MiR-19a-3p overexpression inhibits endothelial dysfunction by targeting JCAD

**Fig. 6:**
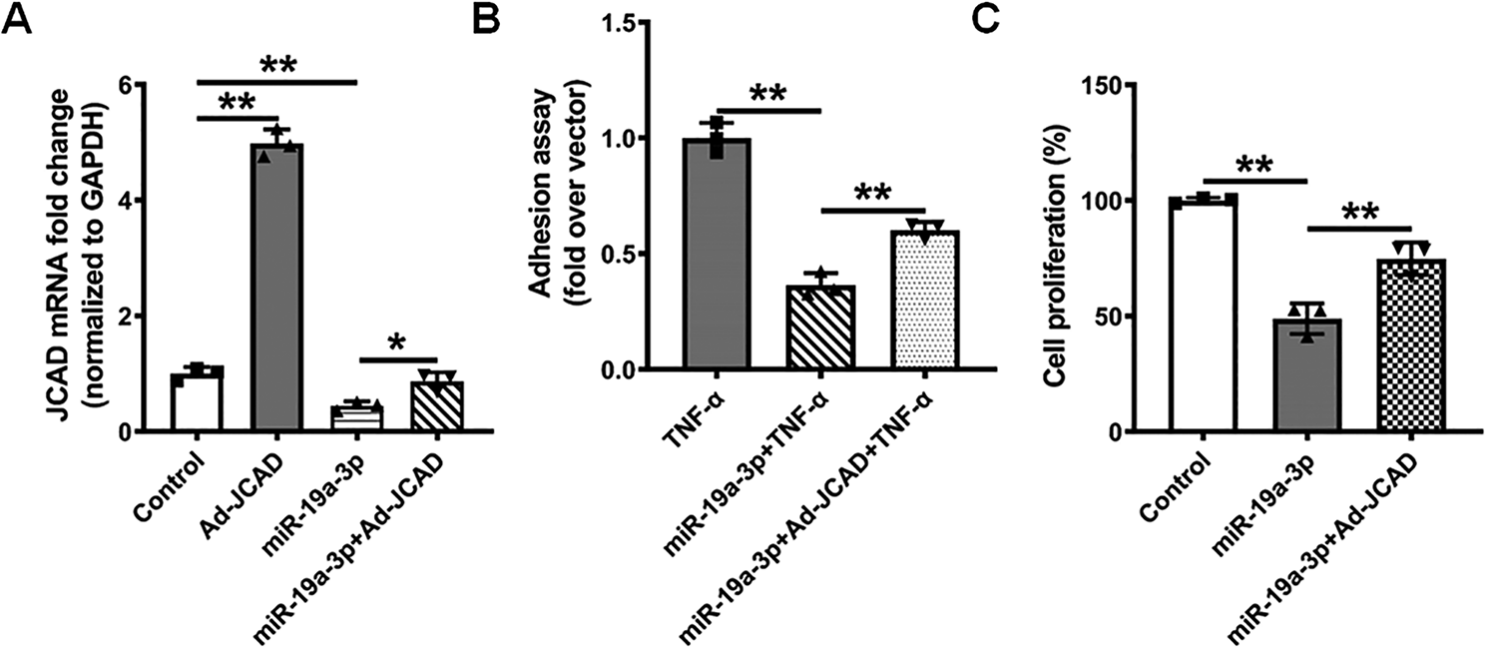
MiR-19a-3p overexpression inhibits endothelial dysfunction by targeting JCAD. (**A**) HUVECs were transfected with Ad-JCAD, miR-19a-3p mimics, or miR-19a-3p mimic + Ad-JCAD for 24 h, and the mRNA expression of JCAD was measured by qRT‒PCR; the results are presented as the mean ± SD (*n* = 3). (**B**) Quantitative data analysis of THP-1 monocyte adhesion to TNF-α-stimulated HUVECs pretreated with miR-19a-3p mimics and miR-19a-3p mimic + Ad-JCAD for 24 h. Data are presented as the mean ± SD (*n* = 3). (**C**) Endothelial cell proliferation was assessed by a CCK-8 assay. HUVECs were transfected with miR-19a-3p mimics and miR-19a-3p mimic + Ad-JCAD for 24 h, and a cell proliferation assay was performed with a CCK-8 kit. The data are presented as means ± SD (*n* = 3). *P < 0.05, **P < 0.01 (ANOVA)

As miRs function by inhibiting the expression of the genes they target, we aimed to verify whether JCAD was the key target of miR-19a-3p in endothelial function by transfecting HUVECs with Ad-JCAD, miR-19a-3p mimic, and miR-19a-3p mimic + Ad-JCAD. Ad-JCAD transfection upregulated the expression of JCAD in HUVECs and weakened the inhibitory effect of miR-19a-3p on JCAD expression (Fig. 6A). MiR-19a-3p significantly reduced THP-1 monocyte adhesion to HUVECs compared to that in the TNF-α treatment group, while JCAD overexpression reduced the inhibitory effect of miR-19a-3p on monocyte adhesion to HUVECs (Fig. 6B). This finding is consistent with the findings of another study showing that JCAD increased endothelial inflammation and exacerbated endothelial dysfunction [47]. Similarly, when we used a CCK-8 assay to assess the role of the miR-19a-3p/JCAD axis in HUVECs proliferation, we found that JCAD overexpression attenuated the effect of miR-19a-3p on the malignant biological behaviors of HUVECs (Fig. 6C). The results highlighted that the miR-19a-3p/JCAD axis is involved in modulating inflammation and the proliferation of endothelial cells. This axis represents a significant regulatory mechanism in endothelial function and the pathogenesis of atherosclerosis, providing new insights into potential therapeutic strategies.

## Discussion

In this work, we revealed a crucial role for miR-19a-3p in protecting against endothelial dysfunction in atherosclerosis. Our investigation demonstrated the downregulation of miR-19a-3p in both atherosclerotic tissue and atherosclerosis-prone treated endothelial cells. The overexpression of miR-19a-3p protected against atherosclerosis-associated endothelial dysfunction, but the knockdown of miR-19a-3p exacerbated endothelial dysfunction, suggesting that miR-19a-3p has the potential to be a diagnostic and prognostic biomarker of atherosclerosis.

According to previous reports, the circulating miR-17-92 cluster is substantially downregulated in people with coronary artery disease and is highly expressed in human endothelial cells [60]. However, whether miR-19a-3p, a member of the “miR-17-92 cluster”, acts as a protective factor in atherosclerosis is unclear. Several studies have suggested that a reduced miR-19a-3p level is associated with the development of atherosclerosis-related conditions [42, 43], the levels and effects of miR-19a-3p in cardiovascular illness remain unclear due to the lack of definitive research results. Our study provides evidence that miR-19a-3p is an atherosclerosis suppressor in endothelial cells, highlighting miR-19a-3p as a potential therapeutic target for combating endothelial dysfunction and atherosclerosis by modulating gene expression in endothelial cells. Continued investigation into its mechanisms and potential clinical applications could significantly impact cardiovascular disease management.

In the progressive pathology of atherosclerosis, the inflammatory cytokine TNF-α has been demonstrated to play a critical role in the disruption of macrovascular and microvascular circulation, leading to endothelial cell destruction and dysfunction [61, 62]. Shear stress, a fundamental determinant of vascular homeostasis, has also been widely investigated in atherosclerosis. The general consensus is that wall shear stress plays a crucial role in the control of endothelial biology [63]. Therefore, we investigated the molecular mechanisms underlying the effect of miR-19a-3p on TNF-α-induced atherosclerosis and shear stress-induced endothelial cells. Several miRs, such as miR-21 and miR-92a, have been reported to be induced by shear stress [64, 65]. In our study, we discovered that the laminar shear stress-treated HUVECs, which is a recognized representation of “atheroprotective” flow conditions, were shown to have significantly higher levels of miR-19a-3p in our study than the control group. However, after treatment with TNF-α, the miR-19a-3p level drastically decreased. Additionally, the miR-19a-3p mimic increased the expression of miR-19a-3p in HUVECs and inhibited cell inflammation and proliferation. These findings suggested that miR-19a-3p could serve as a valuable diagnostic and prognostic biomarker for atherosclerosis. The therapeutic potential of targeting miR-19a-3p to maintain or restore endothelial function in atherosclerosis warrants further investigation. Moreover, understanding the mechanisms by which miR-19a-3p modulates endothelial responses could provide deeper insights into its protective role in vascular health and disease.

JCAD (also known as KIAA1462) was initially discovered to be a novel component of endothelial cell‒cell junctions. Several studies have reported that JCAD is associated with cardiovascular diseases, including atherosclerosis and arterial thrombosis [47, 66]. JCAD colocalizes with VE-cadherin in endothelial cell junctions, and JCAD deficiency may attenuate endothelial dysfunction and atherogenesis by decreasing the expression of proliferative, antiapoptotic, and proinflammatory genes [47]. Although these recent studies have expanded our knowledge of the potential molecular roles of JCAD, our understanding of the regulation of JCAD expression in endothelial cells remains limited. MiRs are crucial regulators of gene expression and may play a significant role in modulating JCAD expression. However, the specific miR that controls JCAD expression is currently unknown. By adversely affecting Hippo/YAP signaling, JCAD in HUVECs causes endothelial dysfunction and increased proliferation, migration, and angiogenesis [67]. In this work, JCAD was confirmed to be the downstream target of miR-19a-3p in HUVECs. We observed that JCAD overexpression attenuated the effects of miR-19a-3p on the malignant biological behaviors of HUVECs. This indicates that miR-19a-3p exerts its protective effects, at least in part, by regulating JCAD expression. Identifying and characterizing this miR-JCAD interaction could reveal new regulatory mechanisms and therapeutic targets for atherosclerosis.

Our results indicated that miR-19a-3p dramatically reduced YAP activity, which is consistent with JCAD deficiency. Additionally, JCAD promoted biological dysfunction in HUVECs and attenuated the inhibitory effect caused by miR-19a-3p overexpression. These findings suggested that miR-19a-3p can attenuate inflammation and the proliferation of endothelial cells by downregulating JCAD. However, it is important to note that miR-19a-3p plays a regulatory role in multiple ways and JCAD is certainly not the only downstream target gene of miR-19a-3p. In subsequent studies, it will be necessary to investigate whether miR-19a-3p can regulate the function of endothelial cells via other mechanisms. In addition, the upstream mechanisms that cause changes in miR-19a-3p expression are worthy of exploration.

There are a few limitations in this study. First, we only assessed the level of miR-19a-3p in TNF-α and laminar flow treated endothelial cells. The influence of other atherosclerotic inducible factors on the expression of miR-19a-3p in endothelial cells was not assessed, and we did not analyze the level of miR-19a-3p in the aortic endothelial cells of atherosclerosis patients due to sample deficiency. Second, we only examined the impact of miR-19a-3p on endothelial dysfunction in a laboratory setting. Future research should include in vivo experiments to validate the findings of this work.

In summary, our data suggest that miR-19a-3p inhibits endothelial dysfunction by targeting JCAD and may act as a protective factor in atherosclerosis. Endothelial cell proliferation and inflammation decreased when miR-19a-3p was overexpressed, resulting in the inhibition of YAP activation. Our work elucidated the important role of the miR-19a-3p/JCAD pathway in endothelial cell functions, providing possible biomarkers and targets for atherosclerosis treatment.

### Electronic supplementary material

Below is the link to the electronic supplementary material.

Supplementary Material 1

## Data Availability

The datasets used and/or analysed during the current study are available from the corresponding author on reasonable request.
