# Supplementary material for: MicroRNA-19a-3p inhibits endothelial dysfunction in atherosclerosis by targeting JCAD
Source: BMC Cardiovasc Disord. 2024 Jul 30;24:394. doi: 10.1186/s12872-024-04063-y (PMC11287888; doi:10.1186/s12872-024-04063-y)
Supplement: Supplementary file 1 — Supplementary Material 1 [file 12872_2024_4063_MOESM1_ESM.docx]

Supplementary Material

# Supplementary Figures
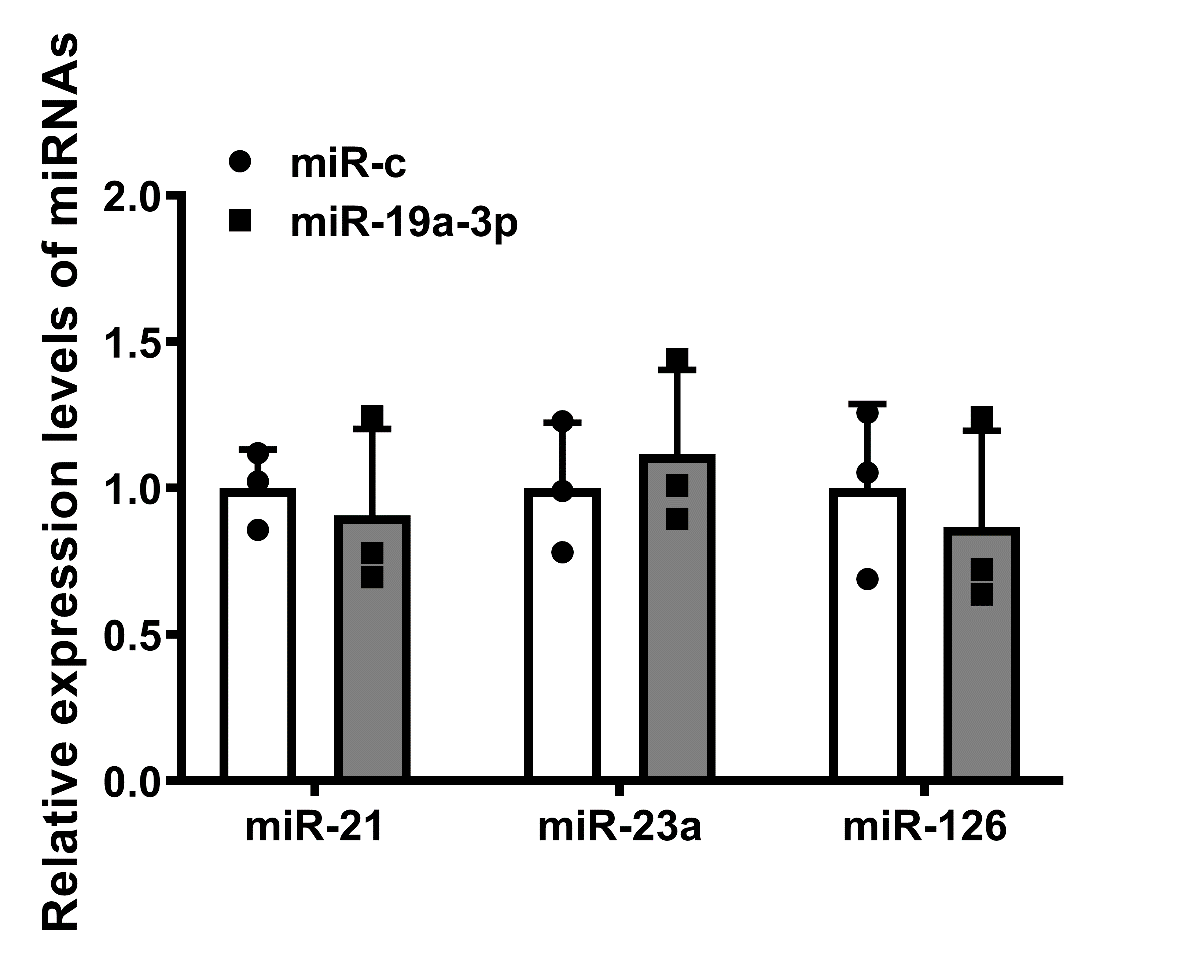


**Supplementary Figure 1.** Overexpression of miR-19a-3p did not influence the expression of these miRNAs. The expression changes of miR-21, miR-23a, and miR-126 in HUVECs after transfection with the miR-c and the miR-19a-3p mimic for 24 h. Data were detected by qRT‒PCR and presented as the mean ± SD (n = 3) (ANOVA).





**Supplementary Figure 1.** Changes in miR-19a-3p levels of the HUVECs at 24 h following transfection with the negative anti-miR control and the anti-miR-19a-3p mimic. Data were detected by qRT‒PCR and presented as the mean ± SD (n = 3). **P < 0.01 (ANOVA).

# Supplementary Tables

**Supplementary Table 1.** Primers sequences.

| Primer for | Primer Sequence 5’ to 3’ |
| --- | --- |
| MiR-19a-3p | Forward: CCTCTGTTAGTTTTGCATAGTTGC |
|  | Reverse: CAGGCCACCATCAGTTTTG |
| MiR-21 | Forward: GCCCGCTAGCTTATCAGACTGATG |
|  | Reverse: GTGCAGGGTCCGAGGT |
| MiR-23a | Forward: CTGGGGTTCCTGGGGATG |
|  | Reverse: GGTCGGTTGGAAATCCCTG |
| MiR-126 | Forward: GCUCGUACCGUGAGTAAT |
|  | Reverse: CAGTGCAGGGT CCGAGGT |
| U6 | Forward: ATTGGAACGATACAGAGAAGATT |
|  | Reverse: GGAACGCTTCACGAATTTG |
| SELE | Forward: GTGTATGTCCTCTGGAGAATGG |
|  | Reverse: GAACCCATTGGCTGGATTTG |
| ICAM1 | Forward: GGCCGGCCAGCTTATACAC |
|  | Reverse: TAGACACTTGAGCTCGGGCA |
| VCAM1 | Forward: TCAGATTGGAGACTCAGTCATGT |
|  | Reverse: ACTCCTCACCTTCCCGCTC |
| JCAD | Forward: CCTGGAACTGGGAATGAGTATG |
|  | Reverse: GTACTGAACGAAGCCGTCATAG |
| CTGF | Forward: GCCCAGACCCAACTATGATTAG |
|  | Reverse: GGAGGCGTTGTCATTGGTAA |
| Cyr61 | Forward: GGCAAGAAATGCAGCAAGAC |
|  | Reverse: CAGTACTTGGGCCGGTATTT |
| GAPDH | Forward: GGAGCGAGATCCCTCCAAAAT |
|  | Reverse: GGCTGTTGTCATACTTCTCATGG |

**Supplementary Table 2.** Source of primary antibodies used for Western blotting.

| **Antibodies** | **Supplier, Cat. No., host** | **Dilution** | **Molecular weight** |
| --- | --- | --- | --- |
| JCAD | Abcam, # ab121545, rabbit | 1: 1, 000 (in 5%BSA) | ~145 kDa |
| CTGF | Santa Cruz, #sc-14939, goat | 1:1, 000 (in 5%BSA) | ~38 kDa |
| Cyr61 | Santa Cruz, #sc-13100, rabbit | 1:1, 000 (in 5%BSA) | ~42 kDa |
| p-YAP (S127) | CST, #13008, rabbit | 1:1, 000 (in 5%BSA) | ~65-78, 54 kDa |
| YAP | Santa Cruz, #sc-15407, rabbit | 1:1, 000 (in 5%BSA) | ~65 kDa |
| α-Tubulin | Huabio, #M1501-1, mouse | 1:10, 000 (in 5%BSA) | ~52 kDa |
